# Outcomes of descemet stripping automated endothelial keratoplasty for pediatric descemet membrane detachment with diffuse corneal edema

**DOI:** 10.3389/fped.2025.1614963

**Published:** 2025-09-02

**Authors:** Hanzhi Ben, Gege Xiao, Pei Zhang, Rongmei Peng, Zijun Xie, Jing Hong

**Affiliations:** Department of Ophthalmology, Peking University Third Hospital, Beijing, China

**Keywords:** descemet stripping automated endothelial keratoplasty, endothelial keratoplasty, pediatric keratoplasty, descemet membrane detachment, forceps-related birth injury

## Abstract

**Purpose:**

To report Descemet stripping automated endothelial keratoplasty (DSAEK) outcomes for pediatric Descemet membrane detachment (DMD) with diffuse corneal edema.

**Methods:**

This study included seven cases of pediatric DMD presented at Peking University Third Hospital during October 2017 and April 2022. The collected data included patient demographics, etiology, configuration of the DMD, preoperative and postoperative vision and central corneal pachymetry, surgical outcomes, and complications.

**Results:**

The mean age of the children was 3.27 ± 4.73 (range: 0.33–13) years old. Etiologies included cataract surgeries, glaucoma surgeries, and forceps-related injuries. In all cases, the central areas of the corneas were involved. Three patients had received Descemetopexy at first but failed. DSAEK was successfully performed in all eyes. Compared to the preoperative visual acuity (LogMAR 2.57 ± 0.23), postoperative visual acuity (LogMAR 0.78 ± 0.25) was significantly improved (*P* < 0.01). Postoperative central corneal pachymetry measured within a month after DSAEK (850 ± 163 μm) showed satisfactory improvement when compared to the preoperative one (1,304 ± 234 μm, *P* = 0.005). Early postoperative complications included graft dislocation in one case and was successfully managed with air bubbling.

**Conclusion:**

Pediatric DMD might suffer a lower success rate of Descemetopexy due to the anatomical peculiarity. Reconstructing visual pathway to promote early visual development justifies more aggressive treatment like DSAEK, which has demonstrated satisfactory results.

## Introduction

Descemet membrane detachment (DMD), namely the separation of Descemet membrane (DM) along with corneal endothelium from the posterior corneal stroma, is a vision-threatening complication that might occur after intraocular surgeries, chemical injuries, and forceps-related ocular injuries (1). The disruption of the fluid transport system of corneal endothelium will lead to diffuse corneal edema and decreased visual acuity. Through the years, exhaustive research has facilitated a thorough knowledge of DMD in the adult group. Researchers have proposed numerous classifications to help decide on treatment (2–5). In most instances, reattachment could be achieved by conservative treatment or Descemetopexy (3, 4, 6–9). However, the literature concerning pediatric DMD remains as yet sparse.

Considering the inability of the young children to express themselves, as well as their poor cooperation in taking ophthalmic examinations, it is hard to detect early pediatric DMD. Small, peripheral DMD may get clinically missed and end up with spontaneous reattachment, while large, central DMD warrant surgical intervention is often delayed until the cornea gets severe edema which finally draws attention. By then, the long-detached Descemet membrane might have developed fibrosis and thus adds to the challenges of the reattachment. Kancherla et.al have once reported a case of DMD caused by forceps-related birth injury in a 5-month-old infant, where the DMD recurred only a month after the previous successful reattachment by Descemetopexy and required a second intervention (10). Corinne Ponchel et.al first reported the use of Descemet stripping automated endothelial keratoplasty (DSAEK) to treat vision loss due to forceps-induced DM tears and detachment in an 8-year-old child (11).

If left untreated, pediatric DMD might lead to progressive corneal decompensation and opacification, thus hampering the development of the neural architecture associated with vision and increasing the risk of amblyopia (12). Therefore, it is imperative to resolve the DMD as soon as detected. We herein present our experience of managing seven cases of pediatric DMD with diffuse corneal edema (including four infants younger than one-year-old). Three of them had initially received Descemetopexy, but the result was unsatisfactory. DSAEK with selective replacement of only the detached DM part appears to be an effective approach, for it could promptly establish proper visual pathway and prevent the development of amblyopia.

## Methods

### Study design

This retrospective study included seven children with DMD seeking medical attention at the Department of Ophthalmology, Peking University Third Hospital, between October 2017 and April 2022. All the surgical interventions were performed by the same experienced corneal surgeon (J. H.) under general anesthesia. This study was conducted in accordance with the principles outlined in the Declaration of Helsinki.

### Preoperative evaluation

Detailed ophthalmic examination was required, including visual acuity test (LogMAR visual chart), slit-lamp examination, intraocular pressure measurement, anterior segment optical coherence tomography (AS-OCT) or ultrasound biomicroscope (UBM). Children under six years old underwent the enema of chloral hydrate to help them fall asleep due to their poor cooperation during the examination.

### Surgical technique

The surgical technique of DSAEK was similar to that described in the earlier publication by the authors (13). The donor tissues stored in McCarey Kaufman medium were obtained from the Eye Bank of Peking University Third Hospital. We specifically chose grafts that possessed endothelial cell count greater than 3,300 cells/mm^2^. The donor grafts were prepared using a microkeratome (Moria Surgical, Doylestown, Pennsylvania, USA) on the artificial anterior chamber of the Moria DSAEK system (Moria Surgical, Doylestown, Pennsylvania, USA) before the surgery. All surgeries were performed under general anesthesia. Pupillary constriction was achieved using 0.2% pilocarpine (Bausch&Lomb, Jinan, Shandong, China) eyedrops in phakic eyes preoperatively. The severely edematous recipient epithelium was scraped to provide better visualization. Considering the peculiarities of pediatric eyes (elastic cornea and sclera, shallow anterior chamber, etc.), we chose to create the main tunnel at limbal location to reduce surgical difficulties and donor damage. Cohesive viscoelastic material (Shanghai Haohai Biological Technology Co., Ltd., Shanghai, China) was used to prevent anterior chamber collapse. Although the excessively detached DM could be easily removed, scoring process was still necessary because corneal stromal fibers and peripheral endothelium outside the DMD lesion might be disrupted by brute force. The donor graft was inserted using the suture pull-through technique. The graft was carefully folded with endothelial side inward and pulled into the Busin's glide (Moria Inc., Doylestown, Pennsylvania, USA). A 10-0 suture was placed through the tip of the donor tissue and tied to create a loop. An anterior chamber maintainer was employed after the removal of cohesive viscoelastic material. The suture, alone with the graft, was placed into and pulled through the whole anterior chamber using micro-forceps from the opposite paracentesis wound. The donor lenticule gradually unfolded in the balanced salt solution (Alcon, Fort Worth, Texas, USA). The limbal tunnel was closed by two to four 10-0 nylon sutures. A complete air fill was performed once the donor tissue was centered and in the appropriate orientation. Surgical slit light was used to confirm the adherence of the graft.

### Postoperative treatment

Postoperatively, patients were asked to stay supine for at least four hours. Sedative drugs were administered under the supervision of an anesthesiologist if necessary. Intraocular pressure was closely monitored to enable the timely detection of the pupillary block. Postoperative medical administration included topical use of 1% prednisolone acetate (Allergan Pharmaceuticals Ireland Ltd, Co.Mayo, Ireland), 0.1% Tacrolimus (Senju Pharmaceutical Co., Ltd. Fukusaki Plant, Hyogo, Japan), tobramycin (Tobrex, Alcon Laboratories, Inc., Fort Worth, Texas, USA), and artificial tears (Hycosan, URSAPHARM Arzneimittel GmbH, Saarbrücken, Germany) four times daily. Those previously diagnosed with glaucoma also accepted anti-glaucoma medication. All patients were referred to a pediatric ophthalmologist to receive amblyopia therapy.

### Statistical analysis

Statistical analysis was performed using SPSS 23.0 (SPSS Inc. Chicago, Illinois, USA). *P*-values of less than 0.05 were considered statistically significant. The visual acuity data were converted to the LogMAR scale, with counting fingers (FC), hand motion (HM), light perception (LP), and no light perception (NLP) converted to LogMAR values of 2.1, 2.4, 2.7, and 3.0, respectively.

## Result

**Figure 1:**
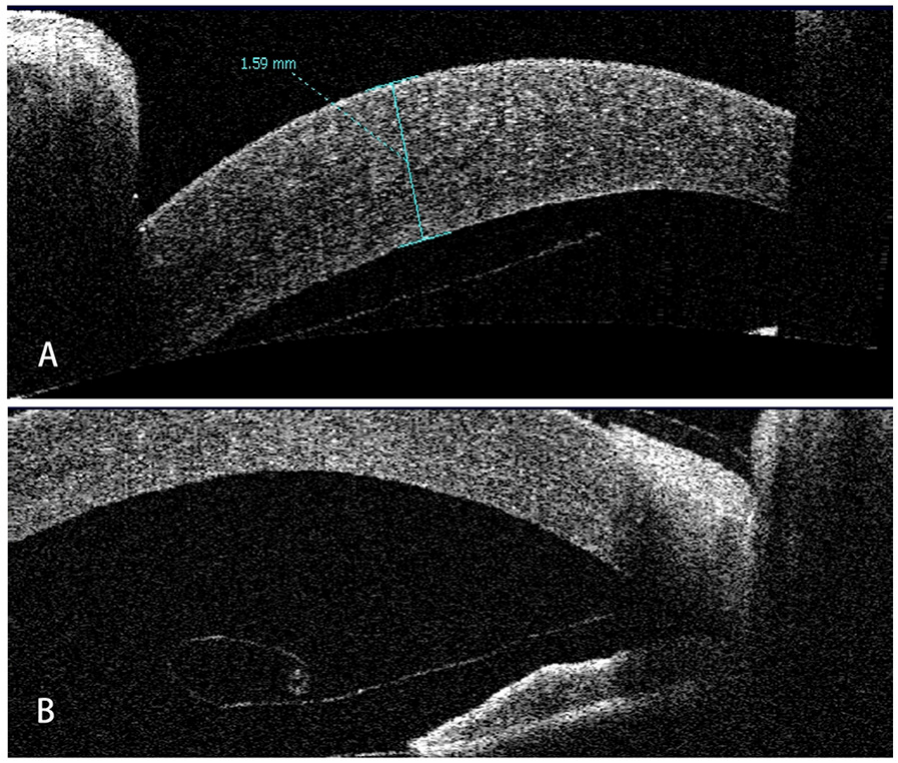
Representative AS-OCT images of pediatric DMD. **(A)** Case 1: tractional DMD with a taut, stretched-out DM. The traction may be caused by the inflammation and fibrosis after cataract surgery. **(B)** Case 2: DMD with scrolled configuration. After receiving Trabeculectomy, Descemet membrane broke off and detached into anterior chamber with the fractured end severely curled up.

Seven children were reviewed in this study, including six boys and a girl. The mean age of the patients was 3.27 ± 4.73 (range: 0.33–13) years old. Five patients developed DMD after intraocular surgeries, of which four had DMD after glaucoma surgery and one following cataract surgery; two patients had DMD secondary to forceps-related birth injury. On average, DSAEK was performed 9.3 ± 13.0 (range: 1–36) weeks after the onset of DMD. Four out of the seven were aphakic eyes. According to AS-OCT or UBM, the central areas of the corneas were involved in all cases. Five had scrolled configuration, and the other two showed taut and stretched-out DM with tractional component (Figure 1). The specific demographics and clinical data are summarized in Table 1.

**Table 1:** The demographic details, preoperative evaluation, surgical treatment and outcomes of the patients.

Case No.	Age/Eye	Etiology	Surgical Intervention	Best Corrected Visual Acuity		CCT		Follow-up (Months)	Comments
				Pre	Post	Pre	Post		
1	6 months/OD	Congenital cataract; ECCE 2 weeks ago	Descemetopexy + DSAEK	2.7	0.82	1,590	860	9	Aphakic eye
2	13 years/OD	JOAG; Trabeculectomy 1 week ago	Descemetopexy + DSAEK	2.1	0.7	1,190	700	29	
3	6 years/OD	PCG; Viscocanalostomy 1 week ago	Descemetopexy + DSAEK	2.4	1.22	970	750	24	Aphakic eye due to congenital cataract (Phaco 5 years ago); combined glaucomatous optic nerve damage
4	21 months/OD	Glaucoma secondary to ECCE (14 months ago); Trabeculectomy 4 months ago	DSAEK	2.7	0.6	1,220	780	64	Aphakic eye
5	6 months/OS	PCG; Trabeculotomy + Trabeculectomy 1 week ago	DSAEK	2.7	1.0	N/A	1,080	53	Aphakic eye due to congenital cataract (Phaco 2 years ago)
6	4 months/OD	Forceps-related birth injury	DSAEK	2.7	0.52	1,550	1,160	36	
7	9 months/OD	Forceps-related birth injury	DSAEK	2.7	0.6	870	N/A	36	

M, male; F, female; ECCE, extracapsular cataract extraction; Phaco, phacoemulsification; JOAG, juvenile open angle glaucoma; PCG, primary congenital glaucoma.

**Figure 2:**
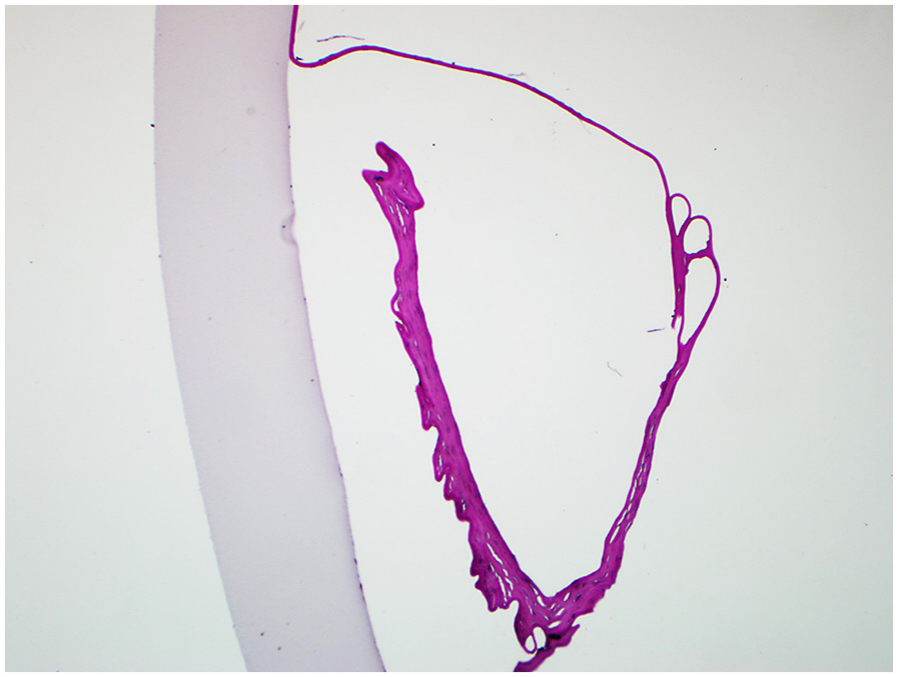
The histological section of the removed DM and endothelium during DSAEK (hematoxylin-eosin stain, original magnification × 40). The detached DM was severely overlapped, forming a large scroll with the endothelium side towards the posterior stroma.

In three cases (Case No. 1–3) where the detachment time was shorter than a month, we first attempted to restore the original DM using Descemetopexy. Evaluation of the anterior chamber using surgical slit-lamp showed successful reattachment intraoperatively. However, in one case (Case No. 2), there had been no improvement in corneal edema, probably due to the endothelial decompensation; in the other two cases (Case No. 1 and 3), AS-OCT revealed that the central DM was detached again in only one week. Thus, DSAEK had to be arranged. Histological examination (hematoxylin-eosin stain) of the tissue removed during DSAEK showed layered DM with scroll formation (Figure 2).

**Figure 3:**
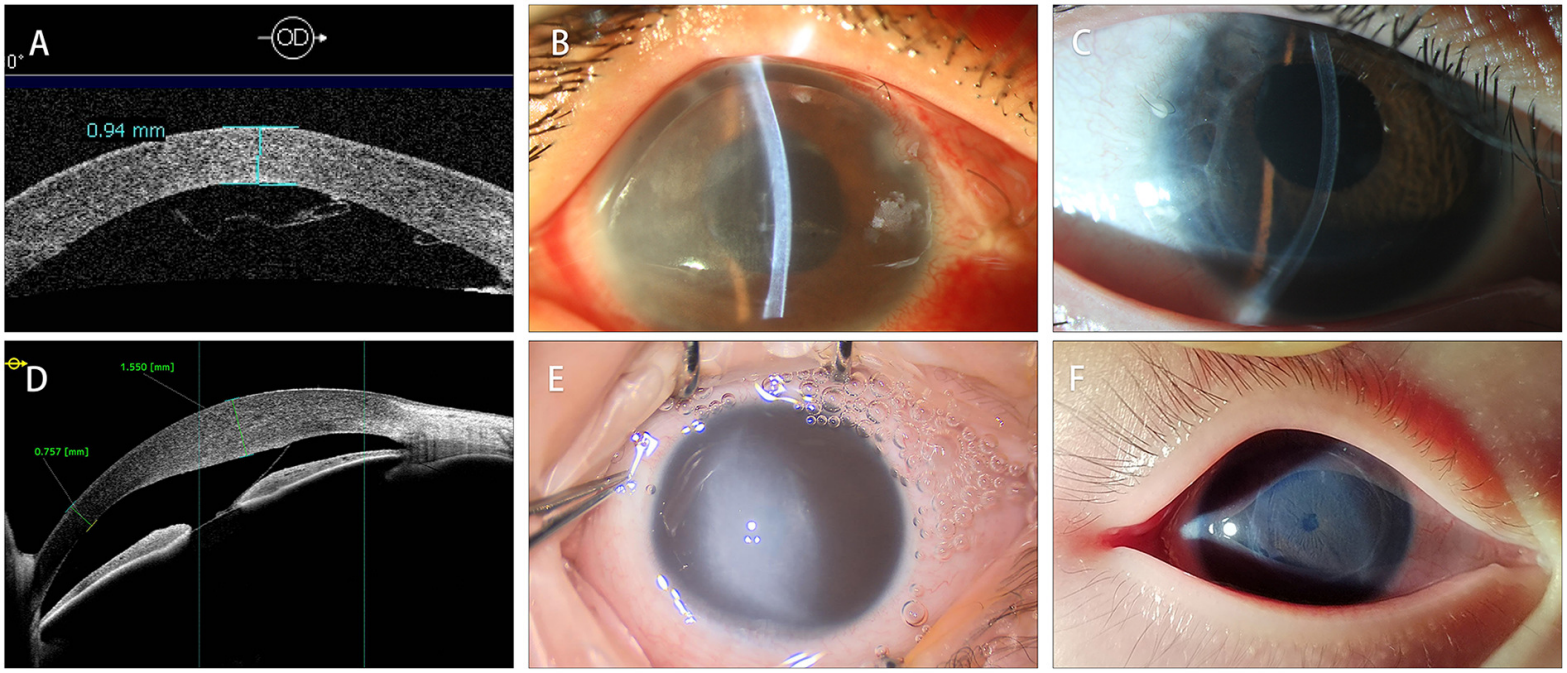
Comparison of preoperative and postoperative corneal condition. **(A)** The AS-OCT of case 3 showed that DM detached and formed scrolls after Viscocanalostomy. **(B)** The clinical photograph of case 3 showed diffused corneal edema. **(C)** The clinical photograph taken two years after the DSAEK showed a well-attached with ideal corneal transparency. **(D)** The AS-OCT of case 6 showed DMD secondary to forceps-related injuries. **(E)** The clinical photograph of case 6. Noticed that the cornea was severely edematous, obscuring the shape of the pupil. **(F)** The clinical photograph taken two years after the DSAEK. The endothelial graft was well-attached with iris texture clearly visible.

Compared to the preoperative visual acuity (LogMAR 2.57 ± 0.23), postoperative best corrected visual acuity (LogMAR 0.78 ± 0.25) was significantly improved (*P* < 0.01) but still limited. The main factors limiting the postoperative visual acuity was ocular comorbidities such as glaucomatous optic nerve damage and aphakic eyes. Postoperative central corneal pachymetry measured within a month after DSAEK (850 ± 163 μm) showed satisfactory improvement when compared to the preoperative one (1,304 ± 234 μm, *P* = 0.005). The surgical success of DSAEK, which was defined as a well-attached DSAEK graft and quick receding of the corneal edema, was achieved in all patients (Figure 3). A slight graft dislocation at the temporal area was detected on AS-OCT two days after the DSAEK in case No.2 and was successfully managed with air bubbling. No other postoperative complication was observed. The average follow-up time after DSAEK is 35.9 ± 18.2 (range: 9–64) months. At the latest follow-up, all patients showed a well-attached DSAEK graft and a clear cornea.

## Discussion

DMD is a noteworthy complication that primarily occurs following intraocular surgeries. With pediatric ophthalmic surgeries booming, the incidence of pediatric DMD is also increasing. It is widely recognized that DMD most commonly occurs after cataract surgery (14, 15). The shallow anterior chamber of children not only increases the difficulty of surgical procedures but also leads to a higher chance of DM exposure and subsequent detachment. In our study, although DMD secondary to glaucoma surgery accounted for more than half of the cases (4/7), most of these children (3/4) had a history of congenital cataract and had received cataract surgery earlier at local hospital. Due to the lack of detailed historical data, it is challenging to determine whether the DMD was caused by the glaucoma surgery alone or the consequence of two surgeries combined. Furthermore, glaucoma-related adverse events also represent serious complications following pediatric cataract surgery (16). Secondary glaucoma following congenital cataract surgery is difficult to manage and often requires surgical intervention (17). It should be noted that Descemetopexy is much less likely to succeed in eyes with a history of glaucoma surgery as the anterior chamber after trabeculectomy or viscocanalostomy is no longer a closed system, making it difficult to maintain an air-filled state (1).

Forceps-related birth injury is also an important etiology of pediatric DMD. The compression of the globe against the orbital roof forcing by the forceps blade can cause varying degrees of DM damage, from the small breaks which could resolved spontaneously to the large tears associated with corneal edema (18). The edges of the torn DM tend to curl toward the stroma, likely due to the difference in elasticity between its anterior banded layer and posterior non-banded layer (19). In past cases, infants with forceps-induced DMD had not been treated promptly and had no choice but to receive penetrating keratoplasty when they grew up (19). Nowadays, ophthalmologists tried to perform DSAEK for these late corneal decompensation adult patients; however, the postoperative visual acuity still remained unsatisfying, mainly due to the severe amblyopia (12, 20). Therefore, early detection and treatment during the infancy is of utmost importance.

Considering the peculiarities of the pediatric ocular anatomy, the management of pediatric DMD might be quite different from the conventional adult DMD. Research has proved that there is a continuous thickening of DM from birth (3 μm) to adulthood (8–10 μm) through the deposition of a nonstriated, nonlamellar material laid down by endothelial cells to the striated prenatal layer (21–23). Twice or three times thinner than the adults', pediatric DM possessed higher elasticity and a greater tendency to shrink and to from scrolls, discovered when researchers started to use donor corneas from children in endothelial keratoplasty. Sonja Heinzelman er.al found that the younger the donor, the longer it would take to unfold the graft in the anterior chamber (24). We ourselves also tried pediatric donor corneas in the previous study and found that endothelial grafts from donors younger than one-year-old tended to develop graft shrinkage during the follow-up (25). This propensity for curling may underlie the scrolled configuration in pediatric DMD.

Descemetopexy has always been regarded as the gold standard surgical treatment for DMD. However, in our study, the Descemetopexy we performed for three patients had all failed, which we had yet to encounter when handling DMD in the adult group. In fact, the percentage of Descemetopexy failure amongst the adult population has decreased to 4.2%–11.5% in recent years (3, 4, 6, 8, 26, 27). Most of the failed cases were prolonged, persistent DMD where DM had formed scrolls or shrunk. Rajat Jain et.al reported the failure of repeat Descemetopexy in a patient with stretched-out DM, while other patients with undeformed DM showed successful reattachment (8). We hence inferred that the scrolled configuration of the DM might be the major factors leading to the failure of Descemetopexy in our study.

Perfluoropropane (C3F8) gas has been used in cases of DMD with scrolled edges or late DMD due to its extended anterior chamber retention time (28, 29). However, in pediatric patients, the critical factor is not the gas retention time but rather the duration for which they can remain supine. In our study, the patients were generally young. Considering cases 4–7 all being infants under two years of age, maintaining the supine position was particularly challenging for them. A fresh DSAEK graft is more likely to adhere to the posterior stroma in a short time compared to the fibrotic old DM with attenuated endothelium. Furthermore, there are also views that suggest 100% air is safe and more efficacious than 14% C3F8 as an agent for descemetopexy (3, 8). On the other hand, repeated attempts at Descemetopexy would delay treatment, not only lead to fibrosis and scarring of the posterior stroma, thereby missing the optimal timing for DSAEK surgery, but also compel multiple exposures to general anesthesia. Therefore, based on our experience with earlier cases, we opted for DSAEK directly in case 4–7.

Penetrating keratoplasty is considered an effective treatment for total or persistent Descemet's membrane detachment (DMD) that is unresponsive to Descemetopexy (1, 30). Given children's strong tissue reactivity and aggressive healing response, the advantages of DSAEK over conventional penetrating keratoplasty (smaller wounds, fewer sutures required, more rapid recovery of vision, lower graft rejection rate, etc.) are especially pronounced in this group (31–33). However, the pediatric anterior chambers are also shallower than the adult's, leading to less operating space and a higher chance for anterior chambers to collapse, thus demanding deft manoeuvres of the surgeon. In this study, all DSAEK grafts were well-attached, and the cornea edema showed significant improvement as early as one week after the keratoplasty. The timely reconstruction of the visual pathway would allow for early amblyopia training and reduce the damage to the visual pathway development.

As the incidence of pediatric DMD increase, while the relevant research remains sparse, our experience of successfully managing these seven cases of severe pediatric DMD may fill the current gap. Nevertheless, we do not suggest that all pediatric cases of DMD necessitate DSAEK. In cases where the DMD is fresh and planar, Descemetopexy still remains a viable and worthwhile option to consider. Central corneal thickness (CCT) may serve as another biomarker for the prediction of recurrent DMD following Descemetopexy, thereby guiding the surgical choice. Studies have demonstrated that greater CCT was associated higher graft detachment rates after Descemet membrane endothelial keratoplasty and the corneal thickness was increased in corneal quadrants with detached grafts compared with adjacent corneal quadrants with attached grafts (34–36). This may be attributable to corneal fibrosis induced by persistent corneal edema, which is particularly pronounced in delayed or forceps-induced DMDs (37).

Furthermore, in 2023, Sharma et.al reported innovative techniques including manual schism combined with Descemetopexy and double bubble pneumo-Descemetopexy, which have demonstrated satisfactory outcome for DMD with scrolled configuration in adult patients (38, 39). These techniques may also be useful in pediatric cases and we intend to explore them in subsequent studies.

The limitation of the study remains its small sample size and retrospective nature. Some of the clinical data such as refraction examination and endothelial cells count were unable to obtain, due to the incorporation of infants for ophthalmic evaluation. Future research should aim to address these limitations by employing larger, prospectively designed studies with more complete clinical data.

## Conclusions

In conclusion, pediatric DMD requires early detection and Individualized management. Compared to adults', pediatric DMD tends to be prolonged DMD with scrolled configuration. The decision and timing of adopting the DSAEK procedure when Descemetopexy failed is of paramount importance for early visual development and the prevention of amblyopia.

## Data Availability

The raw data supporting the conclusions of this article will be made available by the authors, without undue reservation.
